# Dietary Assessment of Shared Plate Eating: A Missing Link

**DOI:** 10.3390/nu11040789

**Published:** 2019-04-05

**Authors:** Tracy Burrows, Clare Collins, Marc Adam, Kerith Duncanson, Megan Rollo

**Affiliations:** 1 School of Health Sciences, Faculty of Health and Medicine, University of Newcastle, Callaghan, NSW 2308, Australia; clare.collins@newcastle.edu.au (C.C.); kerith.duncanson@newcastle.edu.au (K.D.); Megan.rollo@newcastle.edu.au (M.R.); 2 Priority Research Centre for Physical Activity and Nutrition, University of Newcastle, Callaghan, NSW 2308, Australia; marc.adam@newcastle.edu.au; 3 School of Electrical Engineering and Computing, Faculty of Engineering and Built Environment, University of Newcastle, Callaghan, NSW 2308, Australia

**Keywords:** shared plate eating, dietary assessment, lower middle income countries

## Abstract

Shared plate eating is a defining feature of the way food is consumed in some countries and cultures. Food may be portioned to another serving vessel or directly consumed into the mouth from a centralised dish rather than served individually onto a discrete plate for each person. Shared plate eating is common in some low- and lower-middle income countries (LLMIC). The aim of this narrative review was to synthesise research that has reported on the assessment of dietary intake from shared plate eating, investigate specific aspects such as individual portion size or consumption from shared plates and use of technology in order to guide future development work in this area. Variations of shared plate eating that were identified in this review included foods consumed directly from a central dish or shared plate food, served onto additional plates shared by two or more people. In some settings, a hierarchical sharing structure was reported whereby different family members eat in turn from the shared plate. A range of dietary assessment methods have been used in studies assessing shared plate eating with the most common being 24-h recalls. The tools reported as being used to assist in the quantification of food intake from shared plate eating included food photographs, portion size images, line drawings, and the carrying capacity of bread, which is often used rather than utensils. Overall few studies were identified that have assessed and reported on methods to assess shared plate eating, highlighting the identified gap in an area of research that is important in improving understanding of, and redressing dietary inadequacies in LLMIC.

## 1. Introduction

The need to make dietary data more widely available has been reported as one of 10 global research priorities [1]. Access to accurate dietary data relies on use and publication of validated dietary assessment methodologies in a range of settings. Current evidence relating to dietary assessment is focused at the individual level without considering energy and nutrients that may be consumed from shared plate eating. Shared plate eating is an important factor to consider in dietary assessment as it may contribute a substantial proportion of energy and nutrient intake, particularly in those parts of the world where this is how the majority of food is consumed. 

Internationally the way foods and dishes are consumed and the factors that influence consumption vary from region to region [2]. In many high-income countries (HIC) food items are most commonly served on discrete plates for individuals to consume. In other regions dishes are often served centrally with individuals consuming directly from a shared central plate. Shared plate eating has been shown to be evident in many countries, most commonly in Asian countries and low- and lower-middle income countries (LLMIC) [3]. It is likely that the low representation of dietary assessment information relating to shared plate eating is the result of dietary assessment methodology originating in HIC, where shared plate eating is less common.

Assessing dietary intake in low- and lower-middle income countries (LLMIC) is necessary for dietary data to become more available and more applicable to nutrition priorities, but has unique challenges. Compared to high-income countries (HIC), there is substantially less information reported about the way food is prepared, served, and eaten in LLMIC. Details related to how foods are consumed, methods of food preparation, recipes and how nutrient composition data has been compiled and nutrients analysed from the collected intake data and additionally food composition databases are sparse [2,3].

Dietary intake assessment occurs less often in LLMIC and predominantly relied heavily on adaptation of methods used in HIC. For these reasons the data collection methods have been tailored to food consumption from individual plates or servings. Therefore, minimal research attention has focused on shared plate eating where food is directly consumed into the mouth from a centralised dish rather than served individually onto a discrete plate for each person. Shared plate eating is more prevalent and often a defining feature of the way food is often consumed in many LLMIC [4] when compared generally to HIC. When shared plate eating does occur in HIC such as a group sharing pizza or hot chips, it is usually associated with an abundant supply of food. Examples include celebrations or cafeteria, or family-style American meals, all of which differ in context and content from shared meals in LLMIC.

Consuming food in this manner typically occurs multiple times throughout a meal and also when parents are feeding their children. Shared plate eating (sometimes referred to as communal eating) is often overlooked in dietary assessment. Challenges in quantification of shared plate eating include accurate estimation of the number of spoonfuls or handfuls of each dish consumed, the amount eaten from each spoonful/handful, and the highly variable nutrient composition of dishes for which nutrient content have not been characterised or where the composition of each spoonful or handful may vary due to the contents of the dish (for example a meat and vegetable soup where one spoonful may be more liquid based and contain less meat and vegetables and next spoonful may contain more meat and vegetables and less liquid). Additionally, the associated literacy and numeracy skills required by an individual to self-report or for a trained observer to estimate intake from shared plates have not been well described or quantified. 

In addition to the complexity of shared plate eating common in LLMIC, food and nutrient databases are less available compared to HIC [5]. Additional reasons for less dietary intake research being conducted in LLMIC compared to HIC include a lack of context-specific validated dietary assessment tools, low availability of trained personnel to collect and analyse intake data, and limited infrastructure and resources to co-ordinate population-based surveys [5]. 

A review by Ngo et al. 2005 [6] summarised studies that have adapted traditional dietary assessment measures for use in ethnic and/or minority groups, with a specific focus on those of European immigrant groups. The most common dietary assessment methods included in the previous review were interviewer administered food frequency questionnaires (FFQs), 24-h recalls (24HR), and the weighed food record (WFR) [6]. Adaptations to these traditional dietary assessment tools for ethnically diverse groups in LLMIC included identifying key dishes or foods, which may differ from the general population, and determining relevant portion sizes prior to data collection. In addition to the dietary tools, issues also exist with respect to food quantification, limited recipes or unclear recipe construction and lack of inclusion of traditional dishes within nutrient databases [7]. Critical information needed to process dietary data is often also limited or missing in LLMIC, such as country-specific food composition databases and tables of conversion to allow quantification of context-specific portion size [7]. Visual aids have been used in previous studies to assist in quantification of portion size, however it was more common to use standard serving sizes from other studies or countries to quantify intake not population specific [5]. Using pre-defined serving sizes to estimate portion size is likely to incur a bias and incorrect estimation of food intake, especially when the common portion size of dishes in LLMIC are unknown. 

In LLMIC, the use of 24HR has been recommended over other methods such as WFR, due to the perception of being less time consuming and having a lower participant burden [5]. A review of existing dietary assessment in LLMIC identified that while 24HR was most commonly performed using pen and paper, there were substantial costs and burden associated with using this method, particularly the increased time for researchers to code and then analyse data [5]. Unique costs associated within LLMIC have been previously identified and included costs for externally-based researchers to provide training and supervision to upskill research assistants, as well as costs to expand the food composition database, and for logistics (e.g., transportation) and equipment (e.g., internet connections, laptops, mobile phones, phone cards). 

Electronic data capture and use of technology has been suggested as potentially very useful in LLMIC, given that it is likely to be less expensive and more time effective than traditional pen and paper methods [5]. In this way, electronic data capture may overcome some of the identified costs and also potential language/ communication issues. Standardised and streamlined technologies can provide improvements in a range of areas previously acknowledged to improve the ease, time and cost of data collection and processing, and also ensure high-quality standardised data entry, analysis, consistency and comparability across dietary data [7,8].

While externally-based researchers have expertise related to dietary assessment methodologies and understanding of the food supply in their respective countries, it has been identified that identification of foods is more accurate if it involves local people with food expertise [5]. Prynne et al. [5] reported that agreement relating to estimated energy intake by internal and external coders and researchers is quite high, but that at a micronutrient levels understanding of the local food supply and eating habits is essential for more reliable nutrient estimates. 

Research focused on shared plate eating in LLMIC has not been previously reviewed and synthesised, but is important in improving the accuracy of assessing dietary intake in these settings. The aim of the current paper is to provide a narrative synthesis of current research that has reported on the assessment intake from shared plate eating, investigated specific aspects such as individual portion size estimation tools from shared plate eating and use of technology to guide future development work in this area.

## 2. Overview of Research 

The majority of studies that have assessed shared plate eating were undertaken in LLMIC including: Gambia [9] Burkino Faso [10,11,12] and Egypt [13], two in Nepal [14,15], two in India [16,17], Sri Lanka [18] and Zambia [19] with one study each identified as undertaken in Japan and Israel [20] representing higher income countries [21] (Table 1). Studies on shared plate eating were carried out with mothers and children [10,11], children only [14,15,18,19], or adults [9,13,16,17,20,21]. Sample sizes ranged from 17 to 3908. Shared plate eating was found to contribute between 30 and 88% of total daily energy intake [20]. More frequent shared plate eating was reported in rural locations when compared with urban areas [20]. There was no trend identified towards more studies being published in recent years with three studies published in the 1990s and only 3 studies published in 2010 or later.

### 2.1. Variants of Shared Plate Eating

The review identified that different forms of shared plate eating exist, with multiple people eating from one central dish the most common [15]. Others forms of shared plate eating identified included interpolate (or post-serve) sharing defined as two or more people eating from the same plate after serving from a central dish [15]. Food sharing was reported to occur at both meals and snacks and for both adults and children [15].

Shared plate eating was reported to involve complex rules around food distribution based on family structure [22]. For example, an adult male family member may eat first and be offered the protein components of meal first, while women and children will eat from what remains after the men have eaten. This may lead to certain individuals receiving disproportionately less of the food or substantially different meal compositions, and hence varying nutrient intakes at the household level. Further, the feeding of young children differs substantially between households and for children of different ages, which may determine whether a child is self-feeding or being fed by another [23].

### 2.2. Methods of Assessing Dietary Intake 

Dietary assessment methods used to assess intakes from shared plate eating (Table 1) were varied and included 24HR (four studies) [11,12,19,20], two studies that used direct observation [14,15] or food weighing [10,21], and one study using a dietary survey study [17]. Two studies utilised multiple dietary methods; one study used [16] interviews, diet history questionnaire and 24HR while another study used direct observation and ingredient weighing to capture dietary intake [9]. Only one used an objective biomarker, which was doubly-labelled water, to estimate total energy expenditure and to compare energy intake assessed by direct observation [9].

### 2.3. Direct Observation Methods

Direct observation was used in a variety of ways to assess shared plate eating in three studies; two in Nepal [14,15] and one in Gambia [9]. In the study of adult males in Gambia [9] the contribution of two cooked meals per day to energy and nutrient intake of adult males was determined using doubly-labelled water and algorithms based on observation of household food preparation and consumption. The process involved identification and weighing of each ingredient prior to being added to each cooking pot. A researcher observed the preparation process and documented the addition of each ingredient. When the meal was ready for consumption the weight of each empty eating bowl was determined, then weighed again after the addition of the staple (i.e., rice, grains) and again after the addition of each respective meal component. The body weight of each person and the food they consumed from each dish was recorded. The observer remained in the house to weigh any remaining/ leftover food [9].

In the same study the average weights of six common staple foods (rice, sorghum, sanyo, findo, maize, cassava) consumed at each meal and who consumed these foods was determined through direct observation. Estimated intakes for common additions (such as sauces, spices, herbs and condiments) were also determined. Through use of this technique, an algorithm was created to quantify the distribution between individuals of food from shared plate dishes. 

Doubly-labelled water was used to verify total energy intake of adult males, with urine collected over a period of ten days. The results indicated that estimation from two cooked meals was equivalent to 80% of an individual’s total energy expenditure, with the remainder likely to be contributed by snacks between meals which were not assessed in the study. As data collection occurred periodically throughout the year, distinct seasonal changes in the total energy intakes consumed and associated weight status were reported. Higher energy intakes and weight status were reported from October to April coinciding with and following the harvest season in Gambia, and showed a steady decline in middle months of the year. 

The accuracy of visual estimations of children’s food intake during shared plate eating compared to individual-plate eating scenarios was investigated by Shankar et al, 2001 [14] in a study involving male and female Nepali children. In this study, eight trained observers estimated food portions consumed by children enacting common eating scenarios. Test foods were selected from food groups regularly eaten in this region (grains, vegetables, pulses, fruits, meats, dairy, mixed dishes). Foods were weighed at the start of the meal as a reference measure to improve estimations by trained observers, and at the end of meal to quantify volumes of leftover food. Foods were categorised by food group and categorised as individual-plate or shared-plate. Observed food weight estimates were compared to actual weights of 69 food portions of children eating alone and 26 portions where children were eating from a shared plate. Analyses revealed that observer estimates of dark green leafy vegetables (141%) and fruits (139%) tended to be overestimated by the trained observers whereas grains and mixed foods (98% and 96%) were closer to weighed method. Overall, food weights under field conditions were highly correlated with actual weights for individual-plate (*r* = 0.89) and less accurately for shared plate eating (*r* = 0.84). Accuracy of estimations was influenced by food weight with greater error associated with food quantities of less than 70 grams. Mothers or primary caretakers were not always present during a child’s meal and therefore may not have observed the portion eaten, which suggests that proxy report for children’s intake is not always suitable in these settings [14]. 

Another direct observation study that involved Nepali children [15] was used to investigate dietary differences between children with Vitamin A deficiency and those who were Vitamin A sufficient. Household intake was recorded, however the observers focused predominantly on child intake. Food was visually estimated by trained observers as amount consumed and amount lost to spillage, with total estimations completed for everyone except the last person eating as these were ascertained by subtraction. Each food consumed was categorised into a group. A code was assigned to each member of the shared plate eating episode and other members who joined the meal but not the shared plate, with a second food specific code used to readily identify shared plate eating. A feeding episode was defined as all food consumed within a 30 min time frame. For a child, the mean number of feeding episodes was 3.9 and, on average 2.6 people, were at a shared eating occasion. A meal was defined as when three or more people were eating. Shared plate eating accounted for 26% of all feeding episodes compared with 14% for interpolate feeding and seven percent classified as post-serve sharing. Children who ate from shared plates ate larger portions, and were more than twice as likely to consume grains, carotenoid rich vegetables, pulses, fruit, dairy, and meat as children eating from an individual plate. Results from this study identified that children in a shared plate eating situation were more likely to eat Vitamin A-rich foods than children eating individually. 

### 2.4. 24-Hour Recalls

Four studies used 24HR to assess dietary intake from shared plate eating [11,12,19,20], each using variations of standard 24HR protocols that were reported as appropriate for the setting and study design. 

A study in West Africa [11] involved assessment of shared plate eating or collective/ communal dishes by a trained field worker. A qualitative recall of all foods consumed during the previous 24 h was administered to women with children aged under five years. Collective/communal dishes were initially identified by the women in the compound, with the woman in charge then providing a complete list of all the ingredients that were used. The number of different ingredients was counted but quantification (i.e., nutrients) of intake was not measured. A food variety score (FVS) and diet diversity score (DDS) were determined based on either the number of different items or food groups that were consumed the day before the survey [11]. The mean FVS was 8.3 ± 2.9 items (range 4 to 20), indicating a low number of different ingredients. The DDS was 5.1 ± 1.7 food groups (range 2 to 10), indicating very basic diets. Market days were taken into consideration relative to when recalls were conducted, as diet diversity scores were higher on market days due to women eating more vegetables, although not a greater food quantity.

A subsequent study also by Savy et al. [12] was conducted in Burkina Faso to compare dietary diversity scores measured over a 1-day and a 3-day period, and to assess their relationships with socio-economic characteristics and the nutritional status of rural African women who eat communally. A single recall interview for the three previous days was conducted, and included a spontaneous description, followed by prompting for forgotten foods. Verification of ingredients in dishes mentioned was then conducted with the woman responsible for food preparation [12]. Food consumed outside the compound was accounted for through prompted questions. A dietary diversity score (DDS), defined as the number of different food groups consumed by each woman over a given reference period, was calculated by researchers. Foods were grouped using a nine-item classification: cereals/roots/tubers; pulses/nuts; vitamin-A-rich fruits/vegetables; other vegetables; other fruits; meat/poultry/fish; eggs; milk/dairy products; oils/fats. Quantification and food frequency were not considered, with the scores used in analysis as discrete quantitative variables and after categorisation into tertiles. The mean DDS was 3.5 for a 1-day recall, and increased to 4.4 when calculated from a 3-day recall (*p* < 0.0001). The DDS calculated from a 1-day recall was higher when a market day occurred during the recall period. Both scores were linked to the sociodemographic and economic characteristics of the women. Women in the lowest DDS tertile calculated from the 1-day recall had a mean BMI of 20.5 and 17.7% of them were underweight, versus 21.6 and 3.5% for those in the highest tertile (*p* < 0.0003 and *p* < 0.0007, respectively). Authors concluded that the DDS calculated from a 1-day dietary recall was suitable for predicting the women’s nutritional status, with market days requiring consideration.

In an Israeli (defined as a HIC) study [20], it was identified that individuals could provide information at the individual level for bread and food served onto an individual plate, but accuracy was not known for eating from a common plate of varying sizes or eating directly from a larger platter. The United States Department of Agriculture (USDA) 24HR recall multiple pass method was modified for trained interviewers to record three eating practices; (i) individual plate (ii) eating from a common plate (small, medium or large) with bread, and (iii) eating directly from a larger platter. As bread is often used as the utensil for eating from common dishes, the ‘carrying capacity of bread’ was quantified for 28 common dishes prior to the 24HR recalls. The average carrying capacity of bread was reported as 1.3 grams of solid/semi-solid food per gram of bread and 1.0 grams liquid dishes per gram of bread [20]. The modified 24HR recalls were completed using photographs as reporting aids for shared plate foods. The photos showed shared plates with different relative portions removed, and participant selected the photograph that was representative of their portion. Portion sizes for individual foods were reported using standard 24HR recall methods. Mean (SE) energy intake was 9648 (276) kilojoules (kJ)/day for men and 8230 (172) kJ/day for women, of which carbohydrates accounted for 63 to 64%. Energy intake to estimated energy requirement (EER) ratios ranged from 0.87 to 0.93 among non-dieters who ate the usual amount on the recall day. The authors concluded that the modified 24HR recall produced plausible estimates of energy and nutrient intakes, comparable to those obtained in other populations. The modified questionnaire was proposed as a model for modifying instruments to quantify individual dietary intake in other populations that practice shared plate eating. 

### 2.5. Weighed and Estimated Record

In a study by Iwaoka [21], Japanese mothers (*n* = 64) who prepared meals for their daughters were asked to weigh and record all the ingredients used for cooking. The mothers reported the proportions of the shared dish and/or food eaten by each household member. Results obtained from data collection by mothers were compared to independently collected, self-reported shared dish consumption by daughters. Mothers were reported to underestimate intake of their daughters when compared to self-reported intake of the daughters for energy intake (kJ), macronutrient contribution and within food types, including rice and soup dishes [21]. Fifty percent of under-reporting by the mothers was attributable to rice, the staple food. 

### 2.6. Dietary Survey

Ferrucci et al. [17] analysed data from 3625 participants in the Indian Health study. The overarching health study included questions specific to household/communal spice and oil intake, acknowledging the nutritional contributions these make to Indian dietary intake. The number of spices consumed was collected via a ‘food preparer questionnaire’. The questionnaire included detailed information on 19 spices and oils, in order to quantify how much was purchased (g or kg/number in household) within a particular timeframe (week/month). The gram weight of spices purchased from markets was known to the population group and was linked to the data on the number and ages of people in the household. To account for the varying amount of food consumed by different age groups, individuals less than five years were counted as 0.7 individuals, 5–12 years as 0.9 of a person unit and individuals greater than age 12 years were counted as 1.0. The total weight per item per household was then divided by the total person units to calculate per capita consumption of the spice. 

### 2.7. Use of Technology in Assessment of Shared Plate Eating

Four identified studies reported on how technology had been modified to account for shared plate eating, or to improve the quantification of shared plate eating [10,16,17,20]. 

A variety of forms of technology were used, three were predominantly for assisting in the collection of dietary intake information. Ferruci et al. [17] and Daniel et al. [16] used a computer based diet questionnaire using software called Interactive Nutrition Assistant- Diet in India Study (NINA-DISH), which was comprised of four components (i) defined questions on frequency and portion size (ii) an open ended section for each meal time (iii) food preparer questionnaire and (iv) 24 h recall. The system includes a user interface, business logic and the database, so that it can be imported to any database with minimal modifications. The inclusion of multiple methods to assess dietary intake, combined with versatile computer software make such methods generalisable to assessment of shared plate eating in other LLMIC.

Prynn et al. [10] used an electronic method for direct entry for coding diet diaries which included shared plate eating and was constructed around the hierarchal food menu structure that allowed easy adaptation to the Gambian food database. This hierarchal structure starts with rice: rice alone, boiled rice mixed with each of the basic five sauces, rice cooked with ground nuts and thin rice porridge. The third level offers each of the preceding rice levels with common additions such as fish or vegetables.

Abu Saad et al. [20] modified the Unities States Department of Agriculture USDA 24HR multiple-pass recall for the three eating practices (i) eating an item as an individual plate (ii) eating from a common plate with bread (iii) eating directly from a larger platter, this tool was initially piloted in 40 locals and results confirmed that individuals could estimate the amount of bread consumed. 

All four of these studies provide evidence of the potential for technology used in dietary assessment in HIC to be adapted for use in assessing shared plate eating in LLMIC.

### 2.8. Tools to Assist in Portion Size Estimation from Shared Plates

A study by Thoradeniya [18] investigated different types of portion size estimation tools used to quantify Asian foods. Small photographs, life photographs, line drawings, and use of utensils as aids were trialed. All aids except utensils correlated with actual intakes of foods, with household utensils found to only be correlated for vegetables (*r* = 0.69, *p* < 0.01). Estimations using line diagrams were the most accurate with correlations of *r* = 0.73 for cereal-based food and *r* = 0.86 for vegetables (*p* < 0.01). Line diagrams also performed well overall, with 64% correct estimations, 18% overestimated and 18.1% underestimated, compared to household utensils with 0.6% correct estimations. Higher accuracy and precision were achieved with small photographs for amorphous foods and line diagrams for non-amorphous foods. The combination of small photographs (for vegetables) and line diagrams (for other foods) achieved a high correlation (*r* = 0.959, *p* ≤ 0.001), percentage correct estimations (68.3%) and low under estimations (19.9%) and over estimations (11.8%) [18].

Jerome et al. [13] collected ethnographic data on food consumption patterns in Egypt where shared plate eating is common. This case study focused on the local cultural rules regarding food distribution and consumption, and associated rules regarding the order of eating and drinking (who eats or drinks first or last) and how food-consumption priorities are assigned. It was acknowledged that it may not be culturally appropriate to collect individual-level dietary intake data in settings where food is served communally to a household, family or extended family, and highlighted the challenges of determining whether everyone ate something from every dish and how much of each item was consumed by each person in shared plate eating. The importance of improving quantification was emphasised, given that shared plate eating is commonplace in the majority of the ‘non-Western world’.

## 3. Discussion

This narrative review identified that studies assessing shared plate eating were predominantly carried out in LLMIC’s in addition to two HIC’s, Israel and Japan. There was a particular focus on mothers and children particularly for reporting of dietary intakes. Overall, there were few studies identified, highlighting the identified gap in research in this area. Considering the publication year of studies reviewed here there were only few studies included published in the last 10 years. The lack of research in this area may be partly attributed to previously identified challenges associated of conducting dietary intake assessment research in LLMIC [5]. Challenges for LLMIC include language, food composition database limitations, unknown nutritional compositions of traditional foods and spices, high biodiversity of staples [24], variable portion sizes, and low access to trained workers familiar with dietary assessment and eating behaviours. 

Most dietary assessment studies to date have been done in HICs where it is more common to eat from discrete or individual plates. Discrete plate eating in comparison to shared plate eating is easier to capture and quantify as individuals are likely to be more aware of what foods, and the amount they are consuming. Shared plate eating is not as frequent in the home setting in HICs where it is more common to serve or be served discrete plates of food for each individual in the household and when eating out. However, with increasing globalisation, including migration, shared plate eating is becoming more widespread. All of these factors contribute to making shared plate eating of high interest in the dietary assessment field.

It was identified that shared plate eating occurred at both meals and snacks [15], although most studies focused on consumption at meal times only. The importance of assessing between-meal dietary intake or across a whole 24 h period was highlighted in a doubly labeled water biomarker study that indicated that snacks accounted for 20% of total energy expenditure [9]. As research into shared plate eating progresses, consideration will need to be given to capturing dietary intake data from snacks, particularly where the eating occasion structure and the form of shared plate eating may vary at different meal occasions. 

A variety of modes of shared plate eating were found to exist including: eating directly from a central dish, placing portions on to discrete plates to be consumed by individuals, or post-plate sharing whereby food from the central dish is placed on a secondary plate that is shared by multiple people. Post-plate shared eating was reported for both adults and children [12]. Therefore, collection of preliminary ethnographic data collection to ascertain the cultural norms about shared plate eating, before embarking on dietary assessment studies is of high importance [2]. Qualitative data analysis will allow for an appropriate dietary method to be selected and modified to ensure the data collected reflects the usual consumption [13]. 

In this review, mothers or the female household members were usually responsible for reporting and quantifying dietary intake data from shared eating episodes [13]. This is likely to be attributed to the mother’s role in in the procurement and preparation of food, the cognitively challenging tasks of estimating foods consumed [13], and the age of children in the included studies, with many being young children under six years old [10,11,14]. However, in situations where the mother is not always at home for eating occasions [14], or when an individual within the commune is responsible for food preparation [9] the mother may not be the most appropriate dietary intake reporter. This could be pre-empted by collection of ethnographic data.

Food from shared plate eating contributed the majority of total daily energy intake in the two included studies that reported energy intake [9,20]. Despite the 24HR method being used in three other studies [6,7,10], the dietary intake data was used for purposes other than calculation of energy and nutrient intake such as food and diet variety. There is considerable potential for shared plate eating data collection to improve in order for more accurate and comparable dietary intake to be obtained and reported. Accurate assessment of shared plate eating is currently limited by difficulty in quantification, particularly when shared dishes vary in nutrient and fluid proportions [20]. Even if a single dish is served the nutrient composition of each portion is likely to be variable, demonstrating the complexity of this area of dietary assessment. 

In all studies, observers or interviewers were reported to have undertaken training from researchers, however the components of the training were not well reported. Training is likely required including how many dishes are served, who is eating from each plate, how many people ate from a particular dish, the serving vessel (hands/ utensils/ breads) and nutrient compositions of each mouthful. A previous review of technology-based dietary assessment tools found that technologies exhibiting substantial practical constraints and a lack of demonstrated feasibility for use in LLMICs [8]. It has been previously recommended that to increase collection of dietary data in LLMICs, development of contextually adaptable, interviewer-administered dietary assessment platform areas would be of benefit. In the studies reviewed in the current paper that utilised technology, it was identified that the purpose was primarily assist in standardizing the collection of dietary information. 

Recommendations apparent from this review for the progression of research to refine the dietary assessment methodology of shared plate eating include:

(1) Consideration of seasonality which influences the availability and dietary contribution of different foods at different times of year, and harvest season may result in a period of more plentiful food supply for several months, usually once per year [9].
(2) For optimal accuracy in dietary intake estimation, consideration should be given to weighing the staple food, as discrepancies in estimation of the staple are likely to account for the majority of overall daily energy discrepancy [21].
(3) Modified 24HR with photographs of shared plate with portions removed may serve as a model for shared plate eating assessment [20], as these may be easier for participants to estimate than photographs of individual portions.
(4) Clearly defined aims are required in order to adequately capture relevant dietary intake data. For example, if calcium consumption is of interest then increased attention to consumption of edible bones is important, or if micronutrients such as sodium are being assessed, condiment and sauce consumption requires more detailed assessment as these can be significant contributors [17].
(5) Combination approaches to portion size estimation are recommended, rather than one tool in isolation from other methods [18].
(6) Consideration if culturally appropriate to evaluate individual dietary intakes and maybe household intake i.e., group level might be in some regions more acceptable. A clearly defined preparation and planning phase with ethnographic data is essential [7].
(7) The use of consistent terminology to describe shared plate eating in published research would be valuable to and further the field of research for regions/areas where shared plate eating is the cultural norm, the method of quantification of shared plate eating should be reported so data can be consolidated across studies where possible. Alternately, if shared plate eating has not been taken into consideration in assessment where it is known to occur, this should be acknowledged a limitation of research.
(8) Less intrusive methods of assessing shared plate eating, compared to direct observation, need to be developed to ensure dietary undertake assessment is undertaken as objectively as possible. Direct observation studies can influence the way people eat, can be prohibitively expensive and can be inaccurate compared to weighed intake [14].

The use of technology as a means of assessing dietary intake has increased in parallel to the development of image-based methods, wearable devices, and online methods of administering dietary assessment tools [25]. As evidenced in this review, the application of such approaches to shared plate eating remain relatively untested with very few studies reviewed in this last 10 years. However, Caswell et al. [19] have reported efficient collection of 24HR data using tailored software on a tablet platform in a rural district in central Zambia. The tool was considered easy to use by trained interviewers without prior nutrition training or computing experience to administer a 24HR to caregivers on dietary intakes of children participating in an efficacy trial. If technology approaches can be to individual dietary-level dietary assessment in similar demographic groups to that reported by Caswell et al. [19], the extension of this into shared plate eating warrants substantial research investment, particularly considering the need for improved dietary intake and nutritional status of populations who engage in shared plate eating [8]. For camera devices there is a need to investigate the acceptability of this approach, as it yet to be established and tested in a range of population groups and different ethnicities. 

## 4. Conclusions

Shared plate eating is a very common food consumption modality, particularly in LLMIC, but is under-represented in dietary assessment literature. Key factors identified as contributing to improved assessment of shared plate eating were accurate assessment of staple food intake and the need for combined approaches to portion size estimation. It is recommended that dietary assessment methods match the cultural context in which data is being collected, and that technology methods be considered to replace direct observation. Progress in the dietary assessment of shared plate eating depends on use of consistent terminology and documentation of the methods used to quantify shared plate eating, so data can be consolidated across studies where possible. 

## Figures and Tables

**Table 1:** Studies investigating shared plate eating in low- and lower-middle income countries (LLMIC) and reported diet, food, or nutrient outcomes.

Author Year Country	Study Design and Setting	Study Population	Participant Number and Gender	Dietary Assessment Method	Validated/Standardised Method	Primary Outcomes
Studies involving direct observation						
Hudson 1995 [9] Gambia	Repeat cross sectional Rural African community	Unclear	Phase 1: 208 ‘sinkiros’ (cooking unit within family structure) Phase 2: 12 families Phase 3: 7 males	Phase 1: All ingredients identified and weighed before being cooked	Direct observation: Each bowl weighed (1) when empty (2) after staple food added (3) after sauce added. Age, sex, body weight and amount of food waste was recorded for each participant. Phase 3: DLW study	1. Detailed observation and measurement of meal preparation to calculate nutrient intake from each meal. 2. Average weights of staple foods/sauces consumed. 3. Energy intake estimations from two main meals
Shankar et al. 1998 [15] Nepal	Case Control Sarlahi district—rural Nepal. 3 village development communities	Children 1-6yrs at risk of Vit A deficiency	162 households (81 case/ 81 control) Gender NR	Direct observation by 10 local Nepalese males trained for 3 months	Direct observation of control and case participants	1. Classification of feeding episodes: no food sharing/shared plate eating/interplate sharing 2. Shared plate vs individual plate eating 3. Average portion sizes 4. Odds of consuming different food groups by feeding type
Shankar et al. 2001 [14] Nepal	Validation study as part of larger longitudinal study Sarlahi District. Rural region of Nepal	Children aged 1–10 years old.	11 (6 male, 5 female) 17 field tests (9 individual plate, 8 shared plate setting)	Direct observation by 8 observers who undertook 3 months of training	Food weighing used as reference to determine accuracy of observers’ visual direct observation of food intake.	1. Accuracy of observations in individual plate eating and shared plate eating 2. Comparison of estimates between observers
Studies using 24 h recall dietary assessment method						
Abu-Saad et al. 2009 [20] Israel	Cross sectional Semi nomadic population in Southern Israel	Healthy 19–82-year-old semi-nomadic adults visiting hospital patients or attending Maternal and Child Health Care clinics	*n* = 451 (149 male, 302 female) >1× 24HR recall. 40 completed 3 × 24HR recalls	Modified USDA 24HR recall conducted by trained interviewers and administered using the multi-pass method	EI calculated using American Food Information Analysis System. Compared EI from 24HR recall with BMR using the Schofield equation	1. Eating patterns 2. Nutrient intakes 3. EI using Schofield vs recall 4. Day to day variation in 3-day results for 40 respondents
Caswell et al. 2015 [19] Zambia	Cross- sectional	Children aged 4–8 not yet enrolled in school	938 (479 male, 459 female)	24 h recall conducted on tablet by local interviewers	Nutrient intakes were calculated using food composition tables developed for Zambia by HarvestPlus. USDA National Nutrient Database and other local food composition tables.	1. Demographic Characteristics 2. Common foods consumed 3. Nutrient intakes
Savy et al. 2005 [11] 2007 [12] Burkina Faso	Cross sectional	Women living in randomly selected compounds with at least 1 child under 5 years of age.	691 females	Three-day dietary intake 24 h recall conducted by 14 local fieldworkers. Food variety score (FVS) and Dietary Diversity Score (DDS) calculated	NR	1. Relationship FVS + DDS and socio-demographic and economic characteristics 2. Relationships between DDS + FVS and anthropometry 3. Relationship between DDS + FVS and nutritional status
Studies using an interview or questionnaire method						
Daniel et al. 2014 [16] India	Cross sectional 3 regions of India; New Delhi, Mumbai and Trivandrum. Selected to capture cancer registries	Aged 35–69 years old, resided in study area for at least 1 year.	3908 (male and female) completed DHQ, 3862 included in analysis after data cleaning	Interviews conducted by trained staff at home using New Interactive Nutrition Assistant–Diet in India Study of Health (NINA-DISH): (1) DHQ, (2) questions on meal times; (3) food-preparer QA and (4) 24HR recall	NR	1. Number of food items from food groups reported in DHQ & 24HR recall 2. Number of total food items and time taken to complete DHQ & 24HR recall 3. Top food contributors to nutrient values
Ferrucci et al. 2010 [17] India	Cross sectional from national registry (cancer specific content) three regions (New Delhi, Mumbai and Trivandrum)	Aged 35–69 years old, resided in study area for at least one year. Recruited one male and one female/household	3625 (male and female) (New Delhi *n* = 835, Trivandrum *n* = 2,044, Mumbai *n* = 746)	Computer-based diet QA using NINA-DISH software administered by trained field personnel	NR	1. Global spice consumption and cancer incidence 2. Consumption of spices and seasonings in participants 3. Consumption of commonly used cooking oils 4. Socio-demographic characteristics
Iwaoka et al. 2001 [21] Japan	Cohort College	Dietetics students and their mothers	64 females (32 households)	Approximated proportion	Individual-based food weighing method	1. Mean difference energy and nutrient intakes between methods
Studies using dietary assessment tools of interest to shared plate eating						
Jerome 1997 [13] Egypt and Grenada	Case Study NR	Egypt: Kalama village, periurban community. Grenada	NR	Egypt: Household and individual intake, Grenada: Dietary information reported from each individual in the household (not shared plate)	NR	To use both case studies to highlight the importance of matching the dietary assessment method with the culture of the population being studied.
Thoradeniya et al. 2012 [18] Sri Lanka	Cross sectional Laboratory	School children 10–16 years	80 (32 male, 48 female)	Portion size estimation aids of 16 food items: (1) small photographs (*n* = 11 foods, 876 estimations), (2) life-size photographs (*n* = 7 foods, 558 estimations), (3) 2D life-size diagrams (*n* = 16 foods, 1271 estimations) and (4) household utensils (*n* = 6 foods, 475 estimations)	Actual weight of food	1. Precision and accuracy or portion size estimations tools for Asian Countries

Abbreviations: BMR: Basal Metabolic Rate; D: Dimensional; DDS: Diet Diversity Score; DHQ: Diet History Questionnaire; DLW: Doubly-labelled Water; EI: Energy Intake; FVS: Food Variety Score; N: Number; NR: Not Reported; PSEA: Portion Size Estimation Aid; USDA: United States Department of Agriculture.
